# Machine-Learning vs. Expert-Opinion Driven Logistic Regression Modelling for Predicting 30-Day Unplanned Rehospitalisation in Preterm Babies: A Prospective, Population-Based Study (EPIPAGE 2)

**DOI:** 10.3389/fped.2020.585868

**Published:** 2021-02-03

**Authors:** Robert A. Reed, Andrei S. Morgan, Jennifer Zeitlin, Pierre-Henri Jarreau, Héloïse Torchin, Véronique Pierrat, Pierre-Yves Ancel, Babak Khoshnood

**Affiliations:** ^1^Université de Paris, Epidemiology and Statistics Research Center/CRESS, INSERM, INRA, Paris, France; ^2^Elizabeth Garrett Anderson Institute for Womens' Health, University College London (UCL), London, United Kingdom; ^3^SAMU 93, SMUR Pédiatrique, CHI André Gregoire, Groupe Hospitalier Universitaire Paris Seine-Saint-Denis, Assistance Publique des Hôpitaux de Paris, Paris, France; ^4^APHP.5, Service de Médecine et Réanimation Néonatales de Port-Royal, Paris, France; ^5^CHU Lille, Department of Neonatal Medicine, Jeanne de Flandre Lille, France; ^6^Clinical Research Unit, Center for Clinical Investigation P1419, APHP.5, Paris, France

**Keywords:** neonatology, rehospitalisation, prediction, machine-learning, epidemiology

## Abstract

**Introduction:** Preterm babies are a vulnerable population that experience significant short and long-term morbidity. Rehospitalisations constitute an important, potentially modifiable adverse event in this population. Improving the ability of clinicians to identify those patients at the greatest risk of rehospitalisation has the potential to improve outcomes and reduce costs. Machine-learning algorithms can provide potentially advantageous methods of prediction compared to conventional approaches like logistic regression.

**Objective:** To compare two machine-learning methods (least absolute shrinkage and selection operator (LASSO) and random forest) to expert-opinion driven logistic regression modelling for predicting unplanned rehospitalisation within 30 days in a large French cohort of preterm babies.

**Design, Setting and Participants:** This study used data derived exclusively from the population-based prospective cohort study of French preterm babies, EPIPAGE 2. Only those babies discharged home alive and whose parents completed the 1-year survey were eligible for inclusion in our study. All predictive models used a binary outcome, denoting a baby's status for an unplanned rehospitalisation within 30 days of discharge. Predictors included those quantifying clinical, treatment, maternal and socio-demographic factors. The predictive abilities of models constructed using LASSO and random forest algorithms were compared with a traditional logistic regression model. The logistic regression model comprised 10 predictors, selected by expert clinicians, while the LASSO and random forest included 75 predictors. Performance measures were derived using 10-fold cross-validation. Performance was quantified using area under the receiver operator characteristic curve, sensitivity, specificity, Tjur's coefficient of determination and calibration measures.

**Results:** The rate of 30-day unplanned rehospitalisation in the eligible population used to construct the models was 9.1% (95% CI 8.2–10.1) (350/3,841). The random forest model demonstrated both an improved AUROC (0.65; 95% CI 0.59–0.7; *p* = 0.03) and specificity vs. logistic regression (AUROC 0.57; 95% CI 0.51–0.62, *p* = 0.04). The LASSO performed similarly (AUROC 0.59; 95% CI 0.53–0.65; *p* = 0.68) to logistic regression.

**Conclusions:** Compared to an expert-specified logistic regression model, random forest offered improved prediction of 30-day unplanned rehospitalisation in preterm babies. However, all models offered relatively low levels of predictive ability, regardless of modelling method.

## Introduction

Preterm babies experience significant short and long-term morbidity (1, 2) and rehospitalisations constitute an important, potentially modifiable adverse event. Predictive models for rehospitalisation can potentially improve outcomes and reduce care costs (3–5). Models with high predictive power can facilitate the targeting of high-risk groups and inform discharge and follow-up decisions (6, 7). There is a large body of literature relating to the prediction of rehospitalisation across many different patient groups. Logistic regression has traditionally been used to predict binary outcomes such as rehospitalisation (8, 9). But, deriving models that are highly predictive, validated and use predictors that are both clinically useful and available is challenging (8, 10). To improve prediction, rather than just including additional sets of predictors, researchers are increasingly turning to machine-learning algorithms as alternative methods for constructing models (8, 11, 12). Such algorithms are particularly suited to predicting outcomes in high-dimension data. Traditional modelling processes often carry greater concerns for parameter bias and interpretability as well as model parsimony. On the other hand, machine-learning procedures tend to have limited concern for bias in parameter estimates, and are generally more capable of translating increases in model complexity into greater predictive ability (13, 14). Contrasting a traditional clinician-specified model with a machine-learning approach could provide useful insights into the potential value of adopting machine-learning methods in clinical settings (15, 16).

The least absolute shrinkage and selection operator (LASSO) (17) and random forest (18) represent two established machine-learning approaches. The LASSO is a type of regression analysis that performs regularisation, shrinking some coefficients to zero to improve the accuracy of predictions while reducing complexity. Compared to traditional logistic regression, LASSO can provide variable selection and improve prediction by trading increases in coefficient bias for reductions in variance (19–21). Random forest is a classification algorithm that uses multiple decision trees and bagging to merge predictions across the multiple trees. The advantages of random forest include the efficient consideration of larger predictor sets, a reduced risk of overfitting (22) and an ability to manage non-linear relationships between predictors and predicted probabilities more effectively (23, 24). In the literature, machine-learning methods offer mixed results in terms of rehospitalisation prediction (9, 15, 23–27), and their performance when applied to rehospitalisation among preterm babies appears to be as yet untested.

The primary objective of this study was to compare machine-learning methods (LASSO and random forest) to a logistic regression model specified by clinical experts for predicting unplanned rehospitalisation within 30 days in a population of preterm babies.

## Methods

### Study Design and Population

**Figure 1:**
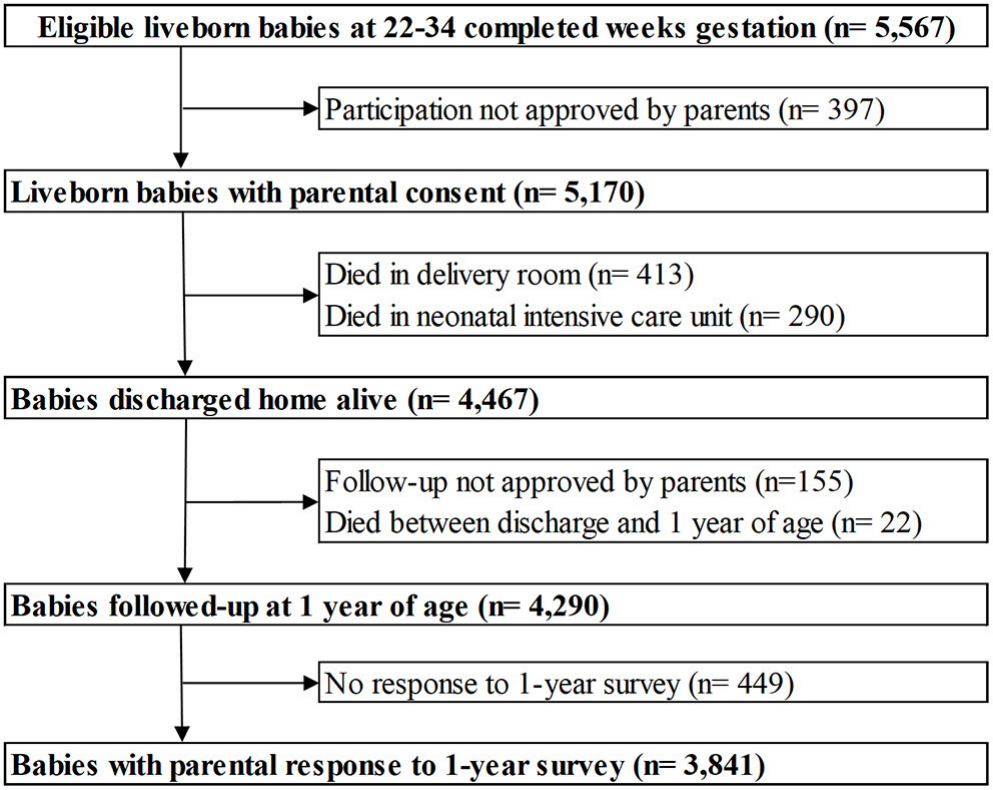
Flowchart of the study population derived from the EPIPAGE 2 cohort.

This study used data from EPIPAGE 2, a French national prospective cohort study of all babies born at 22–34 weeks gestation in all maternity units in 25 French regions between March 28, 2011 and December 31, 2011. The one region that did not participate accounted for approximately 2% of all births in France. Babies with a gestational age of 22–26, 27–31, and 32–24 weeks had recruitment periods of 8 months, 6 months and 5 weeks, respectively (28). Only babies discharged home alive, whose parents completed the 1-year follow-up survey were included in our study. Babies that died during birth hospitalisation or between being discharged and 1-year follow-up were excluded. A flowchart of study sample selection is shown in Figure 1.

The EPIPAGE 2 study included data gathered at birth and via follow-up surveys at 1, 2 and 5.5 years corrected age. Our study used the data collected at birth and the 1-year follow-up exclusively. Birth data were collected using medical records and questionnaires for clinicians in both maternity and neonatal units during the neonatal period. The collection of neonatal data related to a baby's condition at birth, as well as morbidity and treatment status. Data on a mother's socio-economic status, health and their baby's care prior to discharge were collected via interviews and questionnaires in the neonatal unit. The 1-year follow-up questionnaire was sent to parents and gathered information regarding growth, sequela, post-neonatal care, hospitalisations, maternal health and socio-demographics.

### Outcome

Our primary outcome was a binary variable recording whether a baby experienced an unplanned rehospitalisation within 30 days of discharge (URH30). Unplanned rehospitalisation status (URH) was defined according to the rehospitalisation cause recorded in the 1-year follow-up survey. The survey asked parents to provide the date and cause of their baby's three longest rehospitalisations. Selectable causes were bronchiolitis or asthmatic bronchitis, gastroenteritis, diarrhoea or dehydration, poor weight gain, convulsions, injury, malaise, surgery or other (for vaccination or observation for example). We classified rehospitalisations related to vaccinations or surgery as planned, and all other causes as unplanned. Babies experiencing a single rehospitalisation due to both an unplanned and planned cause were classified as having a URH. The number of days between discharge and first URH was calculated to confirm whether a URH occurred within 30 days (URH30). Where a baby experienced multiple URH30s, only the earliest was taken forward into modelling.

### Predictor Variables

All predictor variables were selected from an initial set of 75 EPIPAGE 2 variables (Supplementary Table 1). The first model included 10 predictors and was constructed using logistic regression. Predictor selection for this model was guided by the literature, likely availability in a clinical setting and domain-specific input from expert clinicians. Constructing a parsimonious model with the potential for clinical use was a key priority. This model was a variant of a model we had previously published (29), with continuous predictors replacing their categorical versions. The 10 predictors included in this model were: sex (binary), gestational age in days (continuous), small for gestational age (SGA) status (binary; weight below the 10th percentile for gestational age), receipt of nitric oxide (binary), receipt of surfactant (binary), bronchopulmonary dysplasia (BPD) (30) [categorical; none, mild (≥28 days oxygen and breathing room air to week 36), moderate (≥28 days oxygen and mechanical ventilation or continuous airway pressure/FiO_2_ >21% at week 36) or severe (≥28 days oxygen and mechanical ventilation or continuous airway pressure/FiO_2_ >30% at week 36)], early onset neonatal infection (binary; either no infection or likely infection with antibiotics started at <72 h after birth for ≥5 days or infection confirmed via positive blood or cerebrospinal fluid culture prior to 72 h of life), post-menstrual age at discharge (PMA) in days (continuous), discharge weight in grams (continuous) and breastfeeding status at discharge (categorical; baby in receipt of either no breast milk, mixed feeding or exclusive breastfeeding at discharge).

The remaining two machine-learning models (using LASSO and random forest algorithms) included the full set of 75 predictors. Through this stage of modelling we sought to establish whether machine-learning algorithms, utilising a large number of predictors, could improve prediction.

### Statistical Analysis

The characteristics of babies were compared according to URH30 status using the Kruskal–Wallis test for continuous variables and the chi-squared test or Fisher's exact test for categorical variables. A *p*-value of ≤0.05 was considered statistically significant. All analyses were conducted using R version 4.0.1 (31).

### Predictive Model Building and Validation

All models were initially constructed using complete-cases (babies with no missing values for the outcome or 75 predictors). The first model included 10 predictors, while the second and third included 75 predictors. Optimal hyperparameter values for the LASSO and random forest models were identified via repeated 5-fold cross-validation. The optimal classification threshold for defining events and non-events was established by identifying the value that optimised the true positive and false positive rates (32–35).

The performance of all models was validated through 10-fold cross-validation (36, 37). This randomly divided the data into 10 equally sized subsets. Each time, nine of the subsets were used to train an independent regression model. The derived coefficients were then used to predict on the remaining test subset. This was repeated 10 times so each subset was used as the test once. Model discrimination was assessed using the area under the receiver operating characteristic curve (AUROC), sensitivity, specificity and Tjur's coefficient of determination (38). To establish differences in predictive performance, measures for both the LASSO and random forest models were compared to those for the logistic regression model. DeLong's test (39) was used to identify differences in AUROC and McNemar's test used to compare model sensitivity and specificity. Bootstrapping with 2,000 replications was used to calculate 95% confidence intervals. The Hosmer–Lemeshow goodness-of-fit test and associated calibration curve were used to assess model calibration (40). Additional analyses included scatter plots and partial dependence plots.

### Missing Data

The influence of missing data upon predictive ability was established by rebuilding all models using multiply imputed data (41). Ten imputations and 50 iterations were used for imputation. Further details can be found in our earlier publication using the same dataset (29).

## Results

Of the 5,567 live-born babies eligible for inclusion in the EPIPAGE 2 study; 3,841 were both discharged alive and responded to the 1-year follow-up survey (Figure 1). The rate of 30-day unplanned rehospitalisation in our eligible babies was 9.1% (95% CI 8.2–10.1) (350/3,841). Cross-tabulation of baseline characteristics is shown in Table 1. In the eligible babies, 859 (22.4%) were complete-cases, among whom the rate of URH30 was 9.9% (95% CI 8.0–12.1%) (85/859).

**Table 1:** Distribution of ten primary characteristics of 3,841 eligible babies in the EPIPAGE 2 cohort by 30-day unplanned rehospitalisation (URH30) status.

**Variables**	**Total**	**URH30**	**URH30 (%) (95% CI)**	***p***
**Sex**				
Female	1,818	152	8.4 (7.1–9.7)	
Male	2,001	198	9.9 (8.6–11.2)	0.11
Missing	22	–	–	–
**Gestation age (weeks)**				
32–34	997	40	4.0 (2.8–5.2)	
27–31	2,349	238	10.1 (8.9–11.3)	
22–26	473	72	15.2 (12.0–18.4)	<0.001
Missing	22	–	–	–
**Small for gestational age**				
Yes	1,325	134	10.1 (8.5–11.7)	
No	2,494	216	8.7 (7.6–9.8)	0.16
Missing	22	–	–	–
**Nitric Oxide**				
Yes	163	24	15 (10–20)	
No	3,594	324	9.0 (8.1–9.9)	0.02
Missing	84	–	–	–
**Surfactant**				
Yes	1,888	225	11.9 (10.4–13.4)	
No	1,885	119	6.3 (5.2–7.4)	<0.001
Missing	68	–	–	–
**Early onset neonatal infection**				
Yes	609	61	10 (7.6–12.4)	
No	3,086	275	8.9 (7.9–9.9)	0.43
Missing	146	–	–	–
**Bronchopulmonary dysplasia**				
None	2,915	224	7.7 (6.7–8.7)	
Mild	431	65	15.1 (11.7–18.5)	
Moderate	106	18	17 (10–24)	
Severe	222	30	13.5 (9.0–18.0)	<0.001
Missing	167	–	–	–
**Post–menstrual age at discharge (weeks)**				
<36	639	25	3.9 (2.4–5.4)	
36– <37	960	83	8.6 (6.8–10.4)	
37– <38	763	70	9.2 (7.2–11.3)	
≥38	1,442	172	11.9 (10.2–13.6)	<0.001
Missing	37	–	–	–
**Discharge weight (grams)**				
≤ 2,200	706	46	6.5 (4.7–8.3)	
2,201–2,600	1,484	134	9.0 (7.5–10.5)	
2,601–3,000	977	90	9.2 (7.4–11.0)	
>3,000	579	76	13.1 (10.4–15.9)	0.001
Missing	95	–	–	–
**Breastfeeding status**				
None	1,719	189	11.0 (9.5–12.5)	
Mixed	839	70	8.3 (6.4–10.2)	
Exclusive	1,004	72	7.2 (5.6–8.8)	0.002
Missing	279	–	–	–

*p-Values derived from the chi-squared test*.

### Predictive Model Performance

**Figure 2:**
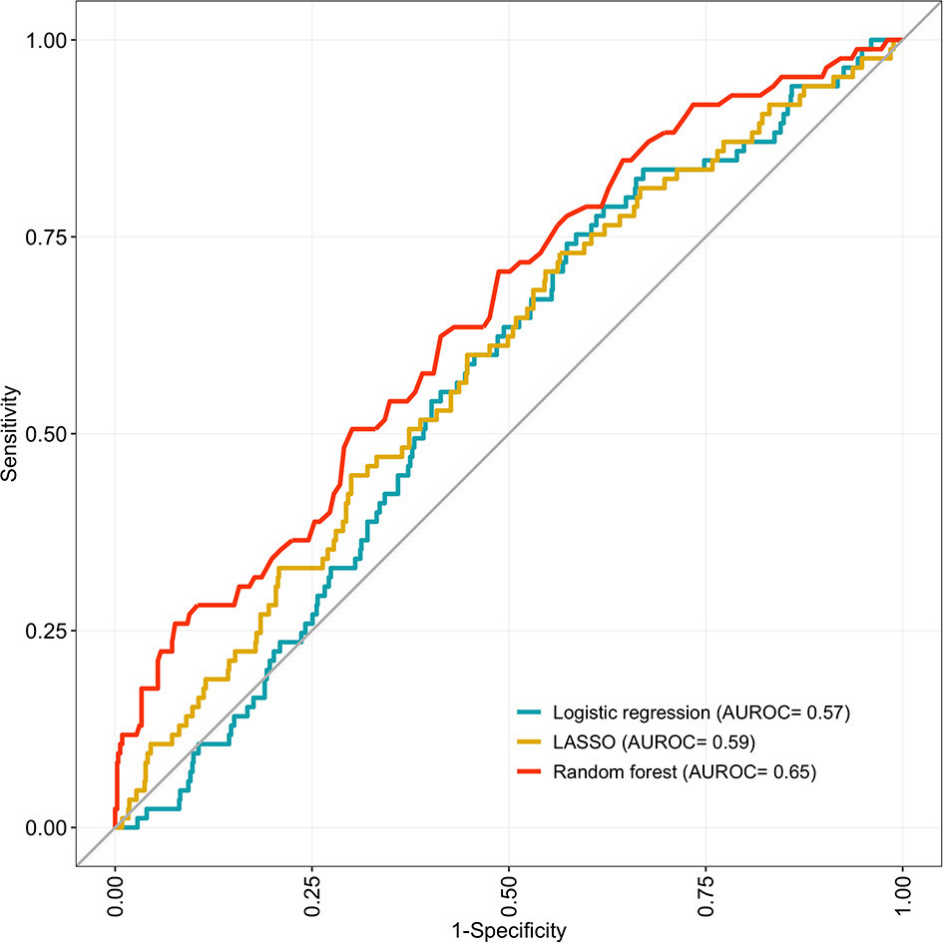
Receiver operating characteristic curves (ROC) for 10-fold cross-validated predictions and corresponding area under the curve (AUROC) for the logistic regression, LASSO and random forest models predicting unplanned rehospitalisation within 30 days. Developed on 859 eligible, complete-case babies in the EPIPAGE 2 cohort and validated using 10-fold cross-validation.

**Figure 3:**
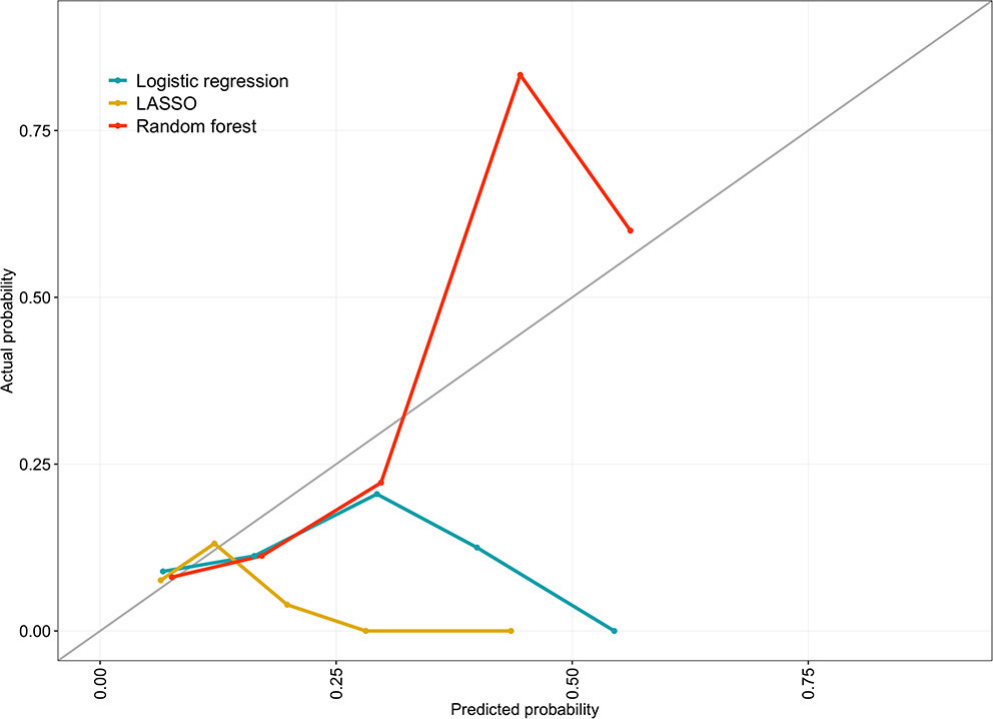
Calibration curve for the logistic regression, LASSO and random forest models comparing the observed probability of unplanned rehospitalisation within 30 days with predicted probability across risk quantiles. Developed on 859 eligible, complete-case babies in the EPIPAGE 2 cohort and validated using 10-fold cross-validation.

The 75-predictor random forest offered a superior AUROC (0.65; 95% CI 0.59–0.71; *p* = 0.03) to the ten-predictor logistic regression (0.57; 95% CI 0.51–0.62). The LASSO model had a similar AUROC (0.59; 95% CI 0.53–0.65; *p* = 0.68) to the logistic regression. All models demonstrated similar sensitivity, but specificity in the random forest (0.59; 95% CI 0.55–0.62; *p* = 0.04) was above that of the logistic regression (0.54; 95% CI 0.51–0.58). The logistic regression and LASSO model demonstrated a significant Hosmer-Lemeshow test statistic. All discrimination and calibration measures for the three models are shown in Table 2, as well as the receiver operating characteristic curves in Figure 2 and calibration curves in Figure 3. Model outputs from the logistic regression are shown in Supplementary Table 2 and a list of the 32 predictors retained by the LASSO model is shown in Supplementary Table 3. Results of additional analyses are shown in Supplementary Figures 1–4.

**Table 2:** Predictive performance measures for the logistic regression, LASSO and random forest models predicting unplanned rehospitalisation within 30 days, constructed on complete-case babies in the EPIPAGE 2 cohort and validated using 10-fold cross-validation.

**Model**	**AUROC (95% CI)**	**Sensitivity (95% CI)**	**Specificity (95% CI)**	**Tjur's coefficient**	**Hosmer-Lemeshow test**
LASSO	0.589 (0.527–0.649)	0.588 (0.482–0.694)	0.553 (0.517–0.587)	0.02	0.02
Random forest	0.648 (0.589–0.710)^*^	0.624 (0.518–0.729)	0.587 (0.552–0.619)^*^	0.06	0.15
Logistic regression	0.565 (0.507–0.620)	0.588 (0.482–0.694)	0.544 (0.509–0.580)	0.01	0.05

*Confidence intervals derived from bootstrap with 2,000 replications*.

* *p ≤ 0.05 in DeLong's test or McNemar's test comparing performance measures to logistic regression*.

Models constructed using multiply imputed data offered similar predictive performance compared to models constructed on complete-cases.

## Discussion

In this study we compared different approaches for the prediction of 30-day unplanned rehospitalisation in preterm babies. We found that the random forest algorithm, constructed on a large and diverse set of predictors, provided improved predictive ability vs. a logistic regression model containing a smaller set of predictors selected by clinical experts. The LASSO algorithm however did not offer improvements over logistic regression. This study produced interesting findings concerning the added value of machine-learning methods such as random forest; contrary to some of the wider literature on clinical prediction models (9, 15, 25).

We propose three reasons that might account for the improved predictions offered by the random forest. Firstly, owing to methods such as “bagging,” random forests are able to retain a greater number of predictors without overfitting (18, 42). Secondly—in reference to our partial dependence plots, which present the marginal effect of a chosen predictor upon the predicted outcome (43)—contrasting the form of the partial dependence plots indicates that the random forest captured non-linear, non-monotonic relationships not seen in the plots for the logistic regression. Assuming such relationships played an important role in determining actual rehospitalisation risk in our sample, then the more effective quantification by the random forest could explain its improved predictive performance. Thirdly, though difficult to confirm conclusively with scatter plots alone, the plots we present suggest that our outcome may be linearly inseparable (where cases and non-cases cannot be split by a straight-line decision boundary). In such situations non-linear algorithms such as random forest can make superior predictions (44–46).

The low predictive ability of all our models fits well within the wider literature on rehospitalisation prediction (8). Even with the random forest providing statistically important improvements in prediction, the limited predictive ability across all the models calls into question the clinical value of such improvements. Model AUROC's ranged from 0.59 to 0.65, indicating that for each model there was an approximately 0.6 probability that a randomly selected rehospitalised baby would be ranked above a non-rehospitalised baby. Specificity in the random forest was superior to logistic regression due to it correctly classifying a greater proportion of true non-cases. Hyperparameter tuning for LASSO identified a relatively small optimal penalty value. Despite this small penalty, a majority of predictors were eliminated from the model (43/75), suggesting they had small coefficients prior to penalisation and were more likely to be uninformative noise variables.

The significant Hosmer-Lemeshow test for the logistic regression and LASSO models indicate that, across quantiles of predicted risk, actual URH30 event counts were not similar to predicted counts. The same test for the random forest provided insufficient evidence to reject the null hypothesis of similar predicted and observed counts. Calibration curves indicate that the models are poorly calibrated. They show that the logistic regression and LASSO models consistently produced predicted probabilities below the actual within quantile URH30 probabilities. Whereas, the random forest provided both inflated and deflated predicted probabilities relative to observed probabilities.

Sensitivity analysis confirmed that missing data did not change the predictive ability of any of the models. There was no difference in predictive ability between any of the models that used complete-case data when compared to the same models using imputed data. The random forest maintained a similar predictive advantage over logistic regression whether modelling was conducted with complete-case or imputed data. As this sensitivity analysis did not reveal an important role for missing data, we chose to present results from the complete-case modelling only. Additional sensitivity analysis revealed that the logistic regression model constructed using a larger set of complete-cases (derived by assessing missingness across the 10 included predictors alone, rather than the maximal set of 75 predictors) did not offer improved predictions.

### Strengths

To the best of our knowledge, this study represents the first comparison of different modelling methods for predicting early rehospitalisation in preterm babies. By contrasting a traditional method that tends to seek interpretable parameter estimates and a balance between complexity and predictive performance, with machine-learning methods that generally have greater capacity to translate complexity into improved predictions, we have addressed a common conflict in the field of clinical predictive modelling.

Our use of data from the EPIPAGE 2 population-based cohort study provided us with a large, representative sample of preterm babies. It also afforded us with a diverse range of clinical, maternal and socio-demographic predictors. Our chosen outcome of 30-day rehospitalisation is a familiar metric of healthcare quality and utilisation. This metric is familiar to clinicians (47, 48) and is well established in the literature on clinical prediction tools (8–10).

Our choice of LASSO and random forest modelling allowed us to assess two established alternatives to logistic regression modelling, each utilising distinct and potentially advantageous methodologies. The LASSO penalises predictor coefficients, providing a form of automated predictor selection that can both reduce over-fitting and optimise classification performance. Random forest on the other hand uses an ensemble of many decision trees, trained by a process of “bagging”; randomly sampling subsets of the training data, fitting the models and then aggregating the predictions. Random forest can also readily capture non-linear relationships in modelling (49, 50). The ability to tune both LASSO and random forest hyperparameters in order to optimise prediction was an additional positive. Tuning of the penalty hyperparameter in LASSO using cross-validation likely reduced the chance that our model eliminated influential predictors.

We included a wide range of performance measures, quantifying two distinct components of prediction (discrimination and calibration). Our use of bootstrap confidence intervals and statistical tests is an additional strength, improving our ability to robustly compare performance between models. Our decision to conduct modelling on both complete-cases and multiply imputed data allowed us to establish the influence of any bias introduced through complete-case analyses. This sensitivity analysis ultimately confirmed that there were no important differences between models constructed using complete-cases and imputed data.

### Limitations

Given our study's focus on predictive ability, model outputs such as effect measures were not a priority concern. However, we acknowledge that the process of coefficient penalisation in LASSO does not deliver interpretable effect measures. Random forest can also be considered a “black-box” method, potentially reducing interpretability and engagement from clinicians (51). For example, the final aggregated tree of a random forest is difficult to interpret given its complexity and often uninterpretable predictor splitting points.

We considered unplanned rehospitalisations to be more preventable than planned rehospitalisations. Our classification of all rehospitalisations for surgery as planned may have meant we excluded unplanned rehospitalisation requiring surgery; alternatively, those babies admitted for planned surgery, may have become infected in hospital and therefore be misclassified as unplanned rehospitalisations. We acknowledge that despite verification against medical records, a mother's recollection of their baby's rehospitalisation may have left our classification of rehospitalisation subject to recall bias. Our choice to exclude babies that died following discharge (0.5%) may also have introduced bias if they tended to have more severe illness, and a greater risk of rehospitalisation within 30 days. Finally, less than 10% of our sample experienced URH30. To address the challenge of constructing predictive models for infrequent events (33, 52, 53) we adopted classification thresholds ranging from 0.9 to 0.11 that optimised the false positive and true positive rates in each model, as recommended in the literature (32–35).

## Conclusion

For the prediction of early unplanned rehospitalisation in preterm babies, a random forest containing 75 predictors provided superior predictive performance compared to an expert-defined logistic regression. However, it is important to acknowledge that while random forest offers improved predictive performance, the extent to which this translates into a clinically valuable increase is uncertain. The failure of LASSO to exceed logistic regression also suggests that the combination of machine-learning algorithms with larger predictor sets is not always sufficient for higher quality predictions. The low predictive performance across all our models suggests that predicting rehospitalisation in preterm babies is complex. Future work should continue to investigate the value of machine-learning methods and also look to identify additional predictors, for example biological markers. Such work might allow better prediction of early unplanned rehospitalisations in preterm babies.

## Data Availability Statement

The data analysed in this study is subject to the following licences/restrictions: Data used in the current study are not publicly available as they contain confidential information but are available from the Scientific Group of the EPIPAGE 2 study for researchers who meet the criteria for access to confidential data on reasonable request. Requests to access these datasets should be directed to EPIPAGE team, epipage.u1153@inserm.fr.

## Ethics Statement

The EPIPAGE 2 study was approved by the National Data Protection Authority (CNIL no. 911009) and by the appropriate national ethics committees (Consultative Committee on the Treatment of Data on Personal Health for Research Purposes—reference: 10.626, Committee for the Protection of People Participating in Biomedical Research—reference: CPP SC-2873). In accordance with national legislation and institutional requirements, written informed consent from the participants' legal guardian/next of kin was not required for the perinatal data collection; all families consented to the ongoing use of their data at the time of 1-year follow-up.

## Author Contributions

RR conceptualised the study, carried out the analyses, and drafted the initial manuscript. BK and AM conceptualised the study, supervised the analyses, and reviewed and revised the manuscript. P-YA conceptualised the study, supervised the analyses, reviewed and revised the manuscript, and was responsible for the overall funding and project administration of the EPIPAGE 2 cohort study. JZ, VP, HT, and P-HJ conceptualised the study and reviewed the manuscript. All authors were involved in manuscript review, read, and approved the final manuscript.

## Conflict of Interest

The authors declare that the research was conducted in the absence of any commercial or financial relationships that could be construed as a potential conflict of interest.

## EPIPAGE 2 Study Group

Alsace: D Astruc, P Kuhn, B Langer, J Matis (Strasbourg), C Ramousset; Aquitaine: X Hernandorena (Bayonne), P Chabanier, L Joly-Pedespan (Bordeaux), MJ Costedoat, A Leguen; Auvergne: B Lecomte, D Lemery, F Vendittelli (Clermont-Ferrand); Basse-Normandie: G Beucher, M Dreyfus, B Guillois (Caen), Y Toure; Bourgogne: A Burguet, S Couvreur, JB Gouyon, P Sagot (Dijon), N Colas; Bretagne: J Sizun (Brest), A Beuchée, P Pladys, F Rouget (Rennes), RP Dupuy (St-Brieuc), D Soupre (Vannes), F Charlot, S Roudaut; Centre: A Favreau, E Saliba (Tours), L Reboul; Champagne-Ardenne: N Bednarek, P Morville (Reims), V Verrière; Franche-Comté: G Thiriez (Besançon), C Balamou; Haute-Normandie: L Marpeau, S Marret (Rouen), C Barbier; Ile-de-France: G Kayem (Colombes), X Durrmeyer (Créteil), M Granier (Evry), M Ayoubi, A Baud, B Carbonne, L Foix L'Hélias, F Goffinet, PH Jarreau, D Mitanchez (Paris), P Boileau (Poissy), L Cornu, R Moras; Languedoc-Roussillon: P Boulot, G Cambonie, H Daudé (Montpellier), A Badessi, N Tsaoussis; Limousin: A Bédu, F Mons (Limoges), C Bahans; Lorraine: MH Binet, J Fresson, JM Hascoët, A Milton, O Morel, R Vieux (Nancy), L Hilpert; Midi-Pyrénées: C Alberge, C Arnaud, C Vayssière (Toulouse), M Baron; Nord-Pas-de-Calais: ML Charkaluk, V Pierrat, D Subtil, P Truffert (Lille), S Akowanou, D Roche; PACA et Corse: C D'Ercole, C Gire, U Simeoni (Marseille), A Bongain (Nice), M Deschamps; Pays de Loire: B Branger (FFRSP), JC Rozé, N Winer (Nantes), V Rouger, C Dupont; Picardie: J Gondry, G Krim (Amiens), B Baby; Rhône-Alpes: M Debeir (Chambéry), O Claris, JC Picaud, S Rubio-Gurung (Lyon), C Cans, A Ego, T Debillon (Grenoble), H Patural (Saint-Etienne), A Rannaud; Guadeloupe: E Janky, A Poulichet, JM Rosenthal (Point à Pitre), E Coliné; Guyane: A Favre (Cayenne), N Joly; Martinique: S Châlons (Fort de France), V Lochelongue; La Réunion: PY Robillard (Saint-Pierre), S Samperiz, D Ramful (Saint-Denis).

Inserm UMR 1153: PY Ancel, V Benhammou, B Blondel, M Bonet, A Brinis, ML Charkaluk, A Coquelin, M Durox, L Foix-L'Hélias, F Goffinet, M Kaminski, G Kayem, B Khoshnood, C Lebeaux, L Marchand-Martin, AS Morgan, V Pierrat, J Rousseau, MJ Saurel-Cubizolles, D Sylla, D Tran, L Vasante-Annamale, J Zeitlin.
