# Supplementary material for: Machine-Learning vs. Expert-Opinion Driven Logistic Regression Modelling for Predicting 30-Day Unplanned Rehospitalisation in Preterm Babies: A Prospective, Population-Based Study (EPIPAGE 2)
Source: Front Pediatr. 2021 Feb 3;8:585868. doi: 10.3389/fped.2020.585868 (PMC7886676; doi:10.3389/fped.2020.585868)
Supplement: Supplementary file 1 [file Data_Sheet_1.PDF]

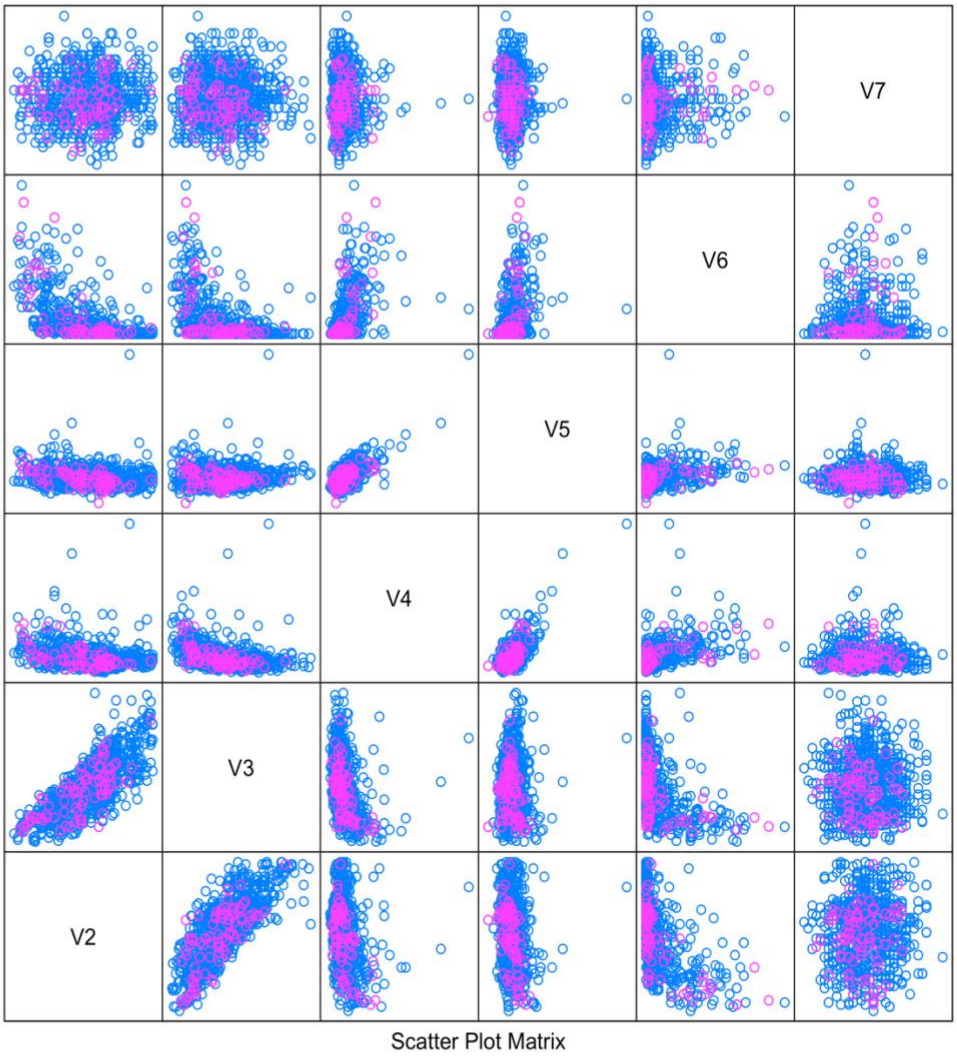

Supplementary Figure 1. Scatter plot matrix of six continuous predictors (gestational age; birth weight; postmenstrual age at discharge; discharge weight; mother's age and days of mechanical ventilation). Pink/Blue = URH30/No URH30
