# Supplementary material for: Machine-Learning vs. Expert-Opinion Driven Logistic Regression Modelling for Predicting 30-Day Unplanned Rehospitalisation in Preterm Babies: A Prospective, Population-Based Study (EPIPAGE 2)
Source: Front Pediatr. 2021 Feb 3;8:585868. doi: 10.3389/fped.2020.585868 (PMC7886676; doi:10.3389/fped.2020.585868)
Supplement: Supplementary file 3 [file Data_Sheet_3.PDF]

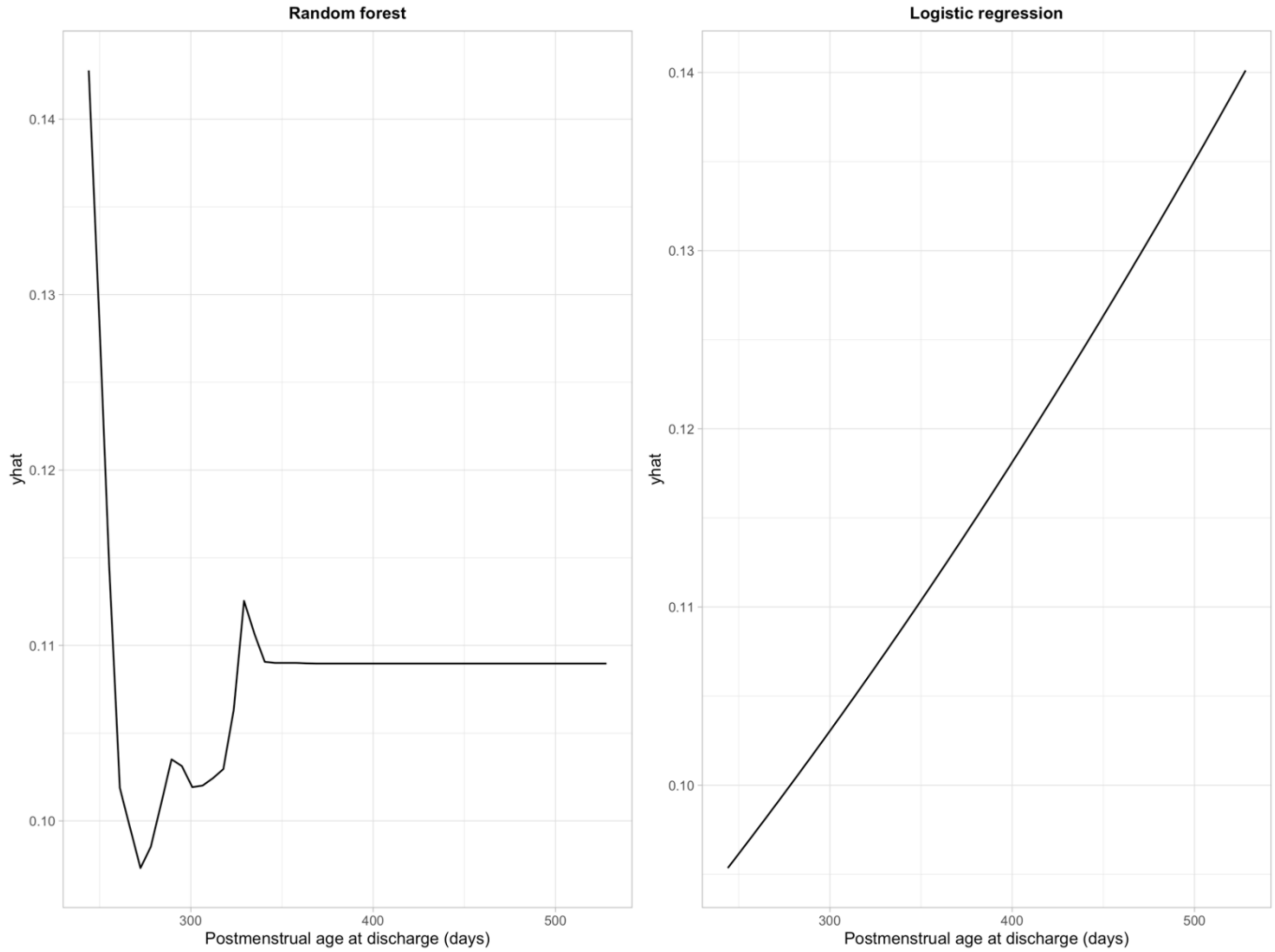

Supplementary Figure 3. Partial dependence plots on postmenstrual age at discharge for the random forest and logistic regression predictive models
